# Guy's Hospital

**Published:** 1829-04-01

**Authors:** 


### GUY'S HOSPITAL.

#### SCIRRHUS OF THE HUMAN MALE BREAST.

An unmarried man, ætatis 30, entered this hospital with a tumour in the right breast as large as one's fist, hard, and irregular. Five years previous to admission, he had met with a blow upon the nipple, and two months afterwards noticed a small, soft tumour, quite unaccompanied with pain. The tumour gradually increased in size, but continued soft for two or three years, when he found it growing harder; after which time it chanced to be exposed to repeated blows, and became the seat of frequent lancinating pains. Five months before his admission, two nipple-like projections appeared, and over these the skin became discoloured as if about to fall into ulceration.

Under these circumstances, Mr. Cooper removed the disease by the knife, when dissection of the tumour disclosed its true nature. A body as large as a walnut, consisting of fibrous lines radiating from a central cartilaginous hardness, formed the nucleus, around which there appeared condensed cellular membrane. The patient bore the operation ill, and the reporter of the case observes that he possessed "a womanly disease, a womanly aspect, and a womanly spirit."

Scirrhus of the male breast is undoubtedly rare, but instances certainly occur now and then. We remember having witnessed a tumour having all the characters of scirrhus in the breast of an octogenarian, who had neither a womanly aspect nor a womanly spirit at all. Considering that at his time of life the disease was not likely to prove of any material detriment, the operation was not recommended by the surgeon.

### GUY'S HOSPITAL.

NON-UNION OF FRACTURED HUMERUS—CURE, TEN MONTHS AFTER THE ACCIDENT, BY MR. AMESBURY'S APPARATUS.

Of all the modes of treating these cases of non-uniting fractures, we fancy that pressure, judiciously applied, will succeed the best, at the slightest expense of pain and danger. Mr. Amesbury deserves credit for the ingenuity displayed in the construction of his various apparatuses for fractured limbs, as well as for the industry he has shewn in collecting the materials of his useful work, lately published, upon fractures. From that work we extract the following case.

John Nickling, twenty-seven years of age, was admitted into Guy's Hospital, under the care of Sir Astley Cooper, on the 17th of July, 1821, with a transverse fracture of the right arm across the middle, occasioned by a blow from a heavy body. A cold lead lotion was applied for a week, and the limb then put up in the usual way, in splints extending from the elbow to the shoulder. After the first week, there was little pain, and the splints were not removed till the end of the sixth, when the bone was discovered not to have united, whilst the broken ends could be rubbed together without producing any pain. The fractured bone was now inclosed in a leathern case, and the limb in a tin trough, bent to a right angle, and reaching from the shoulder to the wrist, the arm being kept supported by a sling. As the muscles wasted, the straps which secured the leather case, were occasionally buckled tighter, in order to keep up strong pressure on the fracture. This plan having been persisted in ten months without success, Sir Astley Cooper examined the limb, and informed the patient that no hope remained, except from an operation. At Mr. Amesbury's request, and Sir Astley's consent, the former gentleman's apparatus was applied, and the man directed to carry the arm in a short sling. The broken ends were pressed together strongly for six weeks, at the end of which time the apparatus was removed, and the bone found firmly united, and as straight as the other. During the treatment, especially at first, the patient had occasionally pains in the fracture, like those of rheumatism, but they gradually subsided.

This was certainly a well-marked instance of the benefits accruing from well-directed pressure, even after a considerable lapse of time. Mr. Amesbury observes, that in cases of non-union of the humerus, when the bone is healthy, and the fractured ends not prevented from uniting by the intervention of any foreign body, he employs the apparatus which he invented for simple fractures of these bones, taking care at the same time to maintain the fractured surfaces "in tight apposition." When the fracture is oblique, this is done principally by padding the apparatus as in recent fractures, according to the direction of the fracture, but when the bone is broken transversely, it is accomplished for the most part by the operation of a short sling, or some other contrivance acting in a line with the long diameter of the bone. We have seen two instances at St. George's Hospital, (Mr. Amesbury candidly states them himself) where that gentleman's method failed, and we cannot but attribute more to constitutional causes in *many* of these cases of non-union, than our author. Indeed, whilst we approve of much that he advances, we cannot but confess that a tone of *brusquerie* is too perceptible in several portions of the work.

